# Looking for Dog Blood Donors in an Endemic Area for Vector-Borne Infections of Central Italy

**DOI:** 10.3390/ani12070817

**Published:** 2022-03-23

**Authors:** Maria Teresa Antognoni, Marta Vascellari, Graziana Da Rold, Federica Toniolo, Sofia Sgubin, Claudia Zanardello, Antonio Carminato, Arianna Miglio

**Affiliations:** 1 Department of Veterinary Medicine, University of Perugia, Via San Costanzo 4, 06126 Perugia, PG, Italy; maria.antognoni@unipg.it; 2 Istituto Zooprofilattico Sperimentale delle Venezie, Viale dell’Università 10, 35020 Legnaro, PD, Italy; gdarold@izsvenezie.it (G.D.R.); ftoniolo@izsvenezie.it (F.T.); ssgubin@izsvenezie.it (S.S.); czanardello@izsvenezie.it (C.Z.); acarminato@izsvenezie.it (A.C.)

**Keywords:** dog blood donors, vector-borne infections, central Italy

## Abstract

**Simple Summary:**

Dogs have proved to be competent reservoir hosts for several vector-borne pathogens, whose prevalence varies according to the area and over time due to the increased movement of people and their pets, climate changes, and vector adaptation strategies. The purpose of this study was to determine the prevalence of some vector-borne pathogens in dog blood donors, living in central Italy. Blood samples of 126 donors included were tested for a broad screening panel for infectious pathogens. The differences in pathogens prevalence according to age, sex, and breeds were tested. Overall, 50 animals tested positive for at least one pathogen. A tendency of hemoplasmas to be more prevalent in older dogs (41.2%) was noted. We highlight the difficulties of selecting healthy blood donor dogs in an endemic area for vector-borne infections. Close collaboration between specialists is important in the interpretation of positive test results. Finally, we underline the important role of blood donors as an epidemiological tool for active surveillance against canine infectious diseases.

**Abstract:**

Dogs are proved to be competent reservoir hosts for several vector-borne pathogens. Their prevalence varies according to the geographical area. Many vector-borne pathogens may be transmitted by blood transfusion. The purpose of this study was to determine the serological and molecular prevalence of some vector-borne pathogens in dog blood donors, living in central Italy. Blood samples of 126 donors (19 breeds) included were tested for a broad serological and DNA-base tests panel. The differences in pathogen prevalence according to age, sex, and breeds were tested (chi-square test, Fisher’s exact test). Overall, 50 animals (39.7%) tested positive at PCR (polymerase chain reaction) and/or serology (IFAT, indirect fluorescent antibody test) for at least one pathogen. Three dogs were positive at both serology and PCR. A tendency of hemoplasmas to be more prevalent in older dogs (41.2%) compared to the younger ones (25.7%) was noted. We highlight the difficulties of selecting healthy blood donor dogs in an endemic area for vector-borne infections. It is important to choose the serological and biomolecular investigations panel that is most suited to the donor’s environment. Close collaboration between clinician and parasitologists is important in the interpretation of IFAT and PCR results. Finally, we underline the important role of blood donors as an epidemiological tool for active surveillance against canine vector-borne diseases.

## 1. Introduction

Blood transfusion is a life-saving treatment in which the recipient intravenously receives the blood of a full matched donor of the same species. In veterinary medicine, the blood transfusion has mutual, even if not balanced, advantages for recipient and donor. Indeed, if the recipient is supposed to benefit from blood transfusions, the donors can indirectly benefit from regular analysis and health status checks [1]. Dogs are proved to be competent reservoir hosts for several vector-borne bacteria and protozoa, transmitted by blood sucking arthropods, including fleas, mosquitoes, sand flies, and ticks [2,3,4]. Moreover, some arthropods are competent vectors for transmission of more than one pathogen. The prevalence of these pathogens varies according to the area and over time [2,3,5,6], due to the increased movement of people and their pets, climate changes, and vector adaptation strategies. In recent years, the availability of a wide spectrum of repellent compounds has significantly improved the prevention of vector-borne infections in dogs [7]. However, the high pressure of vectors and pathogens in certain endemic areas along with the non-correct use of preventative measures may end in multiple and concurrent infections with different pathogens [2,3,4,8,9,10]. In addition, since the majority of canine vector-borne diseases (CVBD) may occur with no or mild clinical, hematological and biochemical abnormalities, many vector-borne pathogens may be transmitted by blood transfusion [3,11]. Due to the variable patterns of disease expression, ranging from subclinical to life threatening infections, the diagnosis of occult CVBDs remains challenging but compulsory for the recruitment of suitable canine blood donors. The international guidelines recommend testing pathogens that meet at least three of the following criteria: (1) the pathogen has been documented to be transmitted by blood transfusion, (2) the pathogen is capable of causing subclinical infection in candidate blood donors, (3) the pathogen can be detected using culture or molecular methods from the blood of an infected animal, and (4) the infection in the recipient has the potential to cause life-threatening illness and be difficult to eliminate with antimicrobial drugs [11].

Central Italy is known to be endemic for several vector-borne pathogens, including bacteria of the genus *Anaplasma*, *Rickettsia*, *Ehrlichia*, and protozoa, such as *Babesia* spp. and *Leishmania infantum* [12,13,14,15,16,17,18,19,20,21,22,23,24].

The purpose of this study was to determine the serological and molecular prevalence of some CVBD pathogens in blood donor candidates, living in central Italy.

## 2. Materials and Methods

Dogs were included in this study if they fulfilled the inclusion criteria based on the Guidelines of the Italian Ministry of Health [25,26] for blood donors: age 2–8 years, body weight ≥ 25 kg, regularly vaccinated and protected against endo- and ecto-parasites as declared by the owners. Sex and breed were also recorded. All the dogs underwent complete clinical examination, hematological and biochemical analysis.

Tripotassium ethylenediaminetetraacetic acid (K3EDTA) anticoagulated blood and serum samples were collected from all dogs to be analyzed by the automated hematology analyzer Sysmex XT-2000iV™ (Sysmex Corp., Kobe, Japan) and the clinical chemistry analyzer (Hitachi 911, Roche, Germany), respectively; cytological examination of blood smears was performed by light microscopy on Wright–Giemsa-stained slides.

### 2.1. Serology

Sera of dogs were tested through immunofluorescence test for Leishmania infantum, Ehrlichia canis, Anaplasma phagocytophilum, and Babesia canis.

The presence of immunoglobulin G (IgG) against *A. phagocytophilum*, *B. canis*, *E. canis* antigens was assessed by indirect fluorescent antibody test (IFAT) using commercial antigens (MegaFLUO^®^, Mega Cor Diagnostik GmbH, Milan, Italy). For the detection of anti-Leishmania IgG, sera were tested with a homemade IFAT following the standard procedures recommended by the Office International des Epizooties and using promastigotes of *L. infantum* zymodeme MON-1 (MHOM/TN/80/IPT-1, Milan, Italy) as a source of antigen. For all the serological tests, commercial anti-canine IgG polyclonal antiserum conjugated to fluorescein isothiocyanate (MegaFluo^®^ FITC IgG, MegaCor Diagnostik GmbH; working dilution 1/100) was used as conjugate. Positive and negative controls provided by the commercial kits were added to each specific reaction for *A. phagocytophilum*, *B. canis*, *E. canis*; positive and negative controls for *L. Infantum* consisted of sera obtained from a cytologically confirmed clinically ill dog, and from a dog that previously tested negative by serological and molecular assays, respectively.

The detection of *Dirofilaria immitis* was performed by the Dirochek^®^ Heartworm Antigen Test Kit (Zoetis Inc., Kalamazoo, MI, USA), an ELISA test capable of detecting circulating serum antigens produced by adult *D. immitis* females.

### 2.2. Molecular Analysis

DNA was extracted from EDTA blood samples using the High Pure PCR Template preparation Kit (Roche Diagnostics, Munich, Germany), according to the manufacturer’s instructions. *L. infantum* detection was performed using in-house SYBR Green Real-Time PCR assay (rPCR) with the primers MC1-MC2 previously described [27]. The reactions were carried out in a total volume of 20 μL, containing 10 μL of QuantiFast SYBR Green PCR Master mix 2X (Qiagen GmbH, Hilden, Germany), 0.1 μM of sense and reverse primers, and 3 μL of extracted DNA. The thermal profile consisted of 5 min of activation at 95 °C, followed by 40 cycles at 95 °C for 15 s, 58 °C for 30 s, and 60 °C for 30 s. Following amplification, a melting curve analysis was performed by slowly raising the temperature of the thermal chamber from 60 to 95 °C. The samples were screened for *Babesia/Theileria* spp. and *E. canis* using rPCR assays performed with the primers and protocols previously described [28] and for *A. phagocytophilum* and *Rickettsia* spp. using rPCR assays performed with the primers and protocols described elsewhere [29]. Hemoplasma were screened using rPCR assay with the primers MycF-MycR1 described elsewhere [30]. The hemoplasma positive samples were amplified with the primers MycE929f-MycE1182r (16S rRNA gene) to allow the identification of the species and rPCR targeting the RNaseP gene was used to distinguish between *Mycoplasma haemofelis* and *Mycoplasma haemocanis* species [30].

To identify the species, the PCR products of good quality were purified and sequenced in both directions using the same forward and reverse primers as sequencing primers in ABI PRISM 3130 Genetic analyzer (Applied Biosystems, Foster City, CA, USA). Nucleotide sequences were compared with representative sequences available in GenBank using Basic Local Alignment Search Tool (BLAST).

In addition, *L. infantum* was searched on a sample of prescapular/popliteal lymph node aspirate from all dogs using the extraction and amplification protocols described above.

### 2.3. Statistical Analysis

The differences in pathogen prevalence between male and female and according to age were tested using the chi-square test or the Fisher’s exact test when appropriate (free software GraphPad available at: https://www.graphpad.com/quickcalcs/contingency2/, accessed on 10 January 2022).

### 2.4. Ethical Statement

Informed consent was obtained from the owners of dog candidate blood donors, as required by the Blood Bank to become eligible donors. The program for donor screening included the collection of information on the health history of the dogs and infectious disease testing as suggested by the Guidelines from the Italian Ministry of Health [25].

## 3. Results

Overall, 126 dogs were included in this study, belonging to 19 different breeds (*n* = 119) (Ariégeois *n* = 19, Bloodhound *n* = 2, Boxer *n* = 1, Italian Bracco *n* = 1, Kurzhaar *n* = 9, Dogo Argentino *n* = 12, American Staffordshire Terrier *n* = 1, Labrador retriever *n* = 5, Greyhound *n* = 1, German Shepherd *n* = 10, Pitbull *n* = 4, Rhodesian Ridgeback *n* = 1, Schnauzer *n* = 1, Giant Hound *n* = 5, Italian Hound *n* = 5, Italian short hair Hound *n* = 3, English Setter *n* = 36, Vandean hound *n* = 2, and Weimaraner *n* = 1) and cross-breeds (*n* = 7). Fifty-seven dogs were female and 69 males, and the average age was 5.3 years (range 2–8 years). The historical information was unremarkable in all the dogs. At clinical examination, 120 dogs were clinically healthy, while 6 dogs showed mild clinical signs, including mild enlargement of 1 or more lymph nodes.

Hematological analysis showed normal CBC values in all but 2 dogs, that showed mild normocytic normochromic anemia. Serum biochemical analysis revealed mild increase in total serum proteins, together with mild hypoalbuminemia and decreased albumin/globulin ratio in 2 dogs, as well as a mild increase in the alanine aminotransferases (ALT 10–40 UI reference range) in 3 dogs. No pathogens were detected by cytological examination of blood smears. All dogs tested negatively for *D. immitis*.

Overall, 50 animals (39.7%) tested positive at PCR and/or serology for at least one pathogen. At serology, 18 dogs (14.3%) tested positive to one of the following pathogens: *L. infantum*, *E. canis*, and *A. phagocytophilum*. At PCR, six dogs (4.8%) were positive for *L. infantum* and 39 dogs (31%) were positive for hemoplasma species. Five dogs were co-infected with *Leishmania infantum* and *Mycoplasma* spp. By PCR

No positivity for *E. canis*, *Babesia/Theileria* spp., *A. phagocytophilum*, and *Rickettsia* spp. was detected in blood samples by PCR. Eleven dogs had antibodies against *L. infantum*, with titles ranging from 1:80 to 1:1280; five of these were positive at PCR, in both blood and lymph nodes, while one dog was positive only in the lymph node. Three dogs were positive at both serology and PCR (dogs N. 2, 3, 40) showed mild clinical signs (i.e., enlargement of one or more lymph nodes) and hemato-biochemical changes, whereas the others were classified as clinically healthy (N.100). Seven out of 11 dogs were co-infected with hemoplasmas, particularly 6 with *Candidatus* M. haematoparvum and 1 with *M. haemocanis*. The details of serological and PCR results, as well as clinical and hematological changes are reported in Table 1 and Table 2.

The prevalence of *L. infantum* and hemoplasmas did not differ significantly according to age and gender, even though a tendency of hemoplasmas to be more prevalent in older dogs (41.2%) with respect to the younger ones (25.7%) was noted (chi-square = 3.334; *p* = 0.0679).

## 4. Discussion

CVB pathogens and vectors are widely present in Italy, particularly in the south and central parts, with high serological prevalence [2,3,5,6,8,13,14,16,17,22,23,24,31,32].

CVBD are insidious for their chronic and subclinical manifestations that could recrudesce, for non-specific clinical signs, and possible coinfections with different pathogens that may complicate the clinical presentation and the diagnostic pathway. Importantly, the clinical and laboratory abnormalities associated with CVBDs, such as vasculitis, hemolytic anemia, thrombocytopenia, and proteinuria, also occur in dogs with idiopathic immune-mediated disease. Since immunosuppression is indicated in the treatment of immune-mediated disease, overlooking infection may significantly affect morbidity and mortality [17]. Finally, vector-borne pathogens should be carefully considered for their zoonotic potential [2,3].

In the field of veterinary transfusion medicine, considering candidate blood donors, the best screening panel for CVBD should be designed according to epidemiological data, dogs and owners’ lifestyles, as well as history and clinical data available. Laboratory investigations should include highly sensitive methods, for different pathogens, in order to rule out occult infections. Moreover, the pathogenic potential of each infectious agent, in both healthy and sick animals, must be considered.

*L. infantum* exposure has spread progressively in the past decades from the endemic southern regions towards northern regions, making the whole Italian Peninsula endemic for this infection [5]. In our study, we observed a seroprevalence of 8.7% for *L. infantum*, that is markedly lower that that reported by a recent study (29.6%), based on the statistical analyses of serological assays performed by two reference diagnostic centers of Italy, over a 10-year period (2009–2019) irrespective of the anamnesis of dogs [5]. It is conceivable that the selection criteria for candidate blood donors, including the lifestyle and the careful use of preventive measures by the owners, may have affected this result, helping in selecting a “low risk” population. Notwithstanding, it is evident that selection criteria and clinical evaluation are not enough to exclude subclinical infection in candidate blood donors. In fact, 6 out of 11 seropositive dogs were also positive in blood and/or lymph nodes by PCR, even if two of them showed low serological titers (1:80). In a previous study, blood samples from 150 privately owned canine candidate blood donors and 338 free-roaming dogs living in northern Italy, were screened by serology for *L. infantum*, *E. canis*, *A. phagocythophilum*, *B. canis*, and *Rickettsia* spp. [28]. In that survey, seroprevalence for *L. infantum* was similar to that registered in the present study, without any significant difference between owned and free-roaming dogs [28]; differently, no PCR positivity was detected in any of the investigated dogs. *L. infantum* has proven to be transmitted by blood transfusion [33], can cause subclinical and chronic disease, has the potential to cause life-threatening illness, and is difficult and long to treat. Thus, blood donations by dogs living or coming from endemic areas require special attention, and the diagnostic approach must include different diagnostic strategies, such as serology and molecular biology. International guidelines for selecting canine blood donors suggest to include only seronegative and blood PCR negative dogs [11]. However, since in endemic areas it may be difficult to select only seronegative blood donors, especially in the case of low antibody titers, caution is required and dogs should undergo a deep clinical and laboratory examination, in order to discriminate among infected dogs that are in a preclinical phase of leishmaniosis and those that only had contact with an infected phlebotomine. The recommendation is to wait and reconsider the dog after a month or two.

Seroprevalences for *E. canis* and *A. phagocytophilum* were 4.0% and 1.6%, respectively, without any PCR positivity. These findings agree with those previously reported by Vascellari et al. (2016) [21], while higher seroprevalences were reported in a survey on stray dogs in southern Italy, with a seroprevalence of 16.03% for *E. canis* and 7.8% for *A. phagocytophilum* [34]. In a previous survey, that considered a large sample of owned dogs living in Central Italy during the period 2013–2017 [13,14], 16.18% seroprevalence for *E. canis* and 3.31% for *A. phagocytophilum* were reported. No statistically significant differences were observed between genders, whereas the highest rate for *E. canis* occurred in animals older than 10 years. Differences among different studies may be due to different management of dogs, particularly in regard to prophylaxis against ticks, as well as to a different prevalence and distribution of tick species in the Italian territory [19,31].

Even though both *E. canis* and *A. phagocytophilum* are primary blood pathogens, affecting canine monocytes and neutrophils, respectively, it seems that PCR blood positivity is more sporadic than for *L. infantum*. According with the ACVIM’s guidelines on canine blood donor screening, the optimal standards should include serology and PCR for both *E. canis* and *A. phagocytophilum* [11]. Regarding *E. canis*, seropositive dogs should not be used as donors, since it is a significant pathogen and it often circulates in monocytes in peripheral blood in low copy numbers and/or intermittently during chronic infection. Thus, negative PCR results cannot rule out the presence of infection in chronically infected dogs [35]. It would be advisable to perform DNA detection tests on tissue samples, other than blood, from animals considered suspicious of having a subclinical *Ehrlichia canis* infection. It could be useful to take tissue biopsies from lymph nodes, blood marrow, and/or spleen as previously suggested and to be performed in chronically infected canines, irrespective of the serological status to *E. canis*, where PCR of DNA extracted from splenic aspirates is reported to be a reliable method for determining the carrier state of Canine Monocytic Ehrlichiosis [36]. Differently, the use of seropositive but PCR negative dogs may be acceptable for *A. phagocytophilum* in areas endemic for *Ixodes* spp., since identification of seronegative donors may be difficult [11]. For both pathogens, seronegative dogs are rarely PCR positive and so serological testing alone could be considered for economic reasons [11]. Although less sensitive, but also cheaper and faster than PCR, cytological examination of blood smears can be helpful in detecting organisms circulating in peripheral blood and should be always included in the screening panel for blood donors. In this regard, it would be important, for each sample, to look at a smear also performed by the buffy coat.

Some authors [33] in the human field, precisely to obviate the danger of transmission of *L. infantum* in endemic areas, recommend the use of leukoreduction during blood collection. This method, which aims to protect the health of the recipient, may be useful also towards *E. canis* and *A. phagocytophilum*. However, considering the costs, this practice in canine blood banking, to date, does not find wide applicability.

Finally, considering lifestyle and breed can also be helpful to determine which diagnostic panel may be more appropriate. For example, hunting or outdoor dogs are at increased risk, particularly for tick-borne diseases, such as *E. canis* and *A. phagocytophilum*.

Unexpectedly, hemoplasmas were the most frequent agent detected in blood of our candidate blood donors, with an overall prevalence of 31.0%. A previous study reported a prevalence of 19.9% of hemoplasma infection, in hunting dogs living in the Campania region (South Italy), without any clinical signs referable to the specific hemoplasma agent detected [37]. A prevalence of 4.5% has been reported in northern Italy, considering a population of candidate blood donors and free-roaming dogs [30]. Interestingly, the prevalence was significantly lower in owned (0.8%) than in free-roaming dogs (6.1%), suggesting that lifestyle could play a role in the risk of infection.

In our study, in fact, most of the hemoplasma positive dogs were involved in hunting (33/39) and only two of these showed slight clinical signs and changes in hematological parameters. Noteworthy, these two dogs were co-infected by *L. infantum*; thus, clinical and hematological changes may be presumably attributed to *L. infantum* infection, rather than hemoplasmas.

In a recent article, sex, age, health status, presence of anemia, and breed were not significantly associated with hemoplasma infection in dogs, but a significant association between hemoplasma infection and other vector-borne pathogens was demonstrated [38]. It is known that dogs can be infected with several hemoplasma species, including *Mycoplasma haemocanis*, “*Candidatus* M. haematoparvum”, and possibly also “*Candidatus* M. haemominutum” [39]. Ticks are suspected to be implicated in transmission even though the mechanism of transmission has not been proven. Since no serologic test is commercially available, diagnosis of hemoplasma infection in routine practice has traditionally been based on cytological examination of blood smears in association with typical clinical symptoms of hemolytic anemia. However, cytological methods have low sensitivity and specificity, particularly in subclinically infected dogs, so PCR is recognized as the gold standard for hemoplasma detection and species differentiation [40]. Even though dogs are generally subclinically infected with these organisms, few cases of hemolytic anemia in splenectomized or immunocompromised dogs have been reported so far [39,41,42]. Only a single clinical infection with “*Candidatus* M. haematoparvum” has been reported in a splenectomized dog even though it was unclear to what extent the hemoplasma played a role in the development of anemia [42].

Therefore, optimally, blood donor dogs should be screened for hemoplasma species by PCR assay and excluded if positive [11]. However, until more is learned about the risk of transfusing hemoplasma positive blood, as well as about the real pathogenetic potential of these organisms, PCR screening could be considered optional, particularly in endemic areas where the availability of blood donors could be significantly restricted due to hemoplasma positivity [11].

## 5. Conclusions

In conclusion, this paper highlighted the difficulties of selecting healthy blood dog donors in an endemic area for vector-borne infections.

As the first step in the selection of blood donors, we recommend a thorough clinical examination, with particular attention to hunting dogs, and a careful evaluation of the blood smear and buffy coat to evaluate the presence of infectious agents. However, considering the existence of subclinical forms, it is important to choose the serological and biomolecular investigation panels that are most suited to the donor’s environment and to the presence of local vector agents. Thus, close collaboration between clinicians and parasitologists is important in the interpretation of IFAT and PCR results, in relation to a pathogen, in order to temporarily or definitively exclude the subject from the donation. Finally, we underline the important role of blood donors as an epidemiological tool for active surveillance against CVBD, that may highlight the circulation of new or neglected pathogens in a specific area and give the opportunity to study the related clinical and hematological signs.

## Figures and Tables

**Table 1:** Number and percentage of dogs that were positive by serology and/or PCR.

Test	*n*. of Positive Dogs	Prevalence (%)
*Leishmania infantum*		
serology	11	8.7
PCR	6	4.8
*Ehrlichia canis*		
serology	5	4.0
PCR	0	0
*Anaplasma phagocytophilum*		
serology	2	1.6
PCR	0	0
*Mycoplasma* spp. PCR	39	31.0
*Candidatus* Mycoplasma haematoparvum	30	23.8
*Mycoplasma haemocanis*	9	7.1
Total		
serology	18	14.3
PCR	45	36.1

**Table 2:** Clinical details of dogs that were positive for *L. infantum* by serology. Results of PCR investigation for *L. infantum* and hemoplasma are reported.

Dog	Age (Years)	Sex	Breed	Clinical Signs	Hematological and Biochemical Changes	*L. infantum* PCR LN	*L. infantum* PCR PB	*L. infantum* IFAT Title	Hemoplasma PCR
1	4	M	Giant hound	healthy	ALT:178 UL	pos	pos	1:320	*Cand.* M. haematoparvum
2	4	M	Giant hound	1 LN enlarged	PT:7.7 g% Alb: 2.84 g% Glob:4.86 g% A/G: 0.58	pos	pos	1:80	*M. haemocanis*
3	4	M	Giant hound	poplitealLN enlarged	ALT: 95 U/L RBC: 4.87 × 10^3^/μL HB: 11.3 g% Ht: 32.5%	pos	pos	1:320	*Cand.* M. haematoparvum
5	5	F	Giant hound	healthy		pos	pos	1:80	*Cand.* M. haematoparvum
23	2	M	English setter	healthy	ALT:54 U/L	neg	neg	1:160	neg
27	5	M	English setter	healthy		pos	neg	1:160	*Cand.* M. haematoparvum
36	2	M	Pitbull	healthy		neg	neg	1:80	neg
40	2	F	Pitbull	popliteal LN enlarged	Pt:10.4 g% Alb: 2.20 g% Glob: 8.2 g% A/G: 0.26 RBC:4.3/mL HB: 8.5 g% Ht: 25.4%	pos	pos	1:1280	neg
72	5	F	Dogo Argentino	healthy		neg	neg	1:80	*Cand.* M. haematoparvum
77	7	M	Crossbreed	healthy		neg	neg	1:80	neg
120	3	M	Dogo Argentino	healthy		neg	neg	1:320	*Cand.* M. haematoparvum

LN = lymph node; F = female; M = male; PB = peripheral blood; ALT = alanine aminotransferase (reference range 10–40 UL); PT = total proteins (r.r. 6.0–7.5 g%); Alb = albumin (r.r. 2.9–3.5 g%); Glob = globulin (r.r. 3.1–4.0 g%); A/G = albumin to globulin ratio (r.r. 0.6–1.1); RBC = red blood cells (5.2–7.9 × 10^3^/μL); HB = hemoglobin (r.r. 12.4–19.2 g%); Ht = hematocrit (r.r. 35–52%).

## Data Availability

Not applicable.
